# Advanced Processing of Giant Kelp (*Macrocystis pyrifera*) for Protein Extraction and Generation of Hydrolysates with Anti-Hypertensive and Antioxidant Activities In Vitro and the Thermal/Ionic Stability of These Compounds

**DOI:** 10.3390/antiox12030775

**Published:** 2023-03-22

**Authors:** Paniz Biparva, Armin Mirzapour-Kouhdasht, Shahriyar Valizadeh, Marco Garcia-Vaquero

**Affiliations:** 1 Department of Food Science and Technology, School of Agriculture, Shiraz University, Shiraz P.O. Box 71441-65186, Iran; 2 School of Agriculture and Food Science, University College Dublin, Belfield, D04 V1W8 Dublin, Ireland; 3 Department of Natural Resources and Environmental Engineering, School of Agriculture, Shiraz University, Shiraz P.O. Box 71441-65186, Iran

**Keywords:** macroalgae, seaweed, ultrasound-assisted extraction, angiotensin-converting enzyme I inhibitory activity (ACE), bioactive peptides

## Abstract

In this study, giant kelp was explored under various conventional and ultrasound-assisted extraction (UAE) conditions for the extraction of protein, its hydrolysis, and ultrafiltration to generate multiple fractions. The amino acid composition of all the fractions and their biological activities in vitro, including angiotensin-converting enzyme I (ACE) inhibitory activity and antioxidant activities (2,2-diphenyl-1-picrylhydrazyl (DPPH) radical scavenging, reducing power (RP), and ferrous chelating (FC) activities) were tested by storing the compounds for 2 weeks at various temperatures (−20–60 °C) and pHs (2–11) to elucidate their thermal and ionic stability, respectively. The yield of protein extraction using the conventional method was lower (≈39%) compared to the use of UAE (150 W, 15 min), which achieved protein recoveries of approximately 60%. After enzymatic hydrolysis and ultrafiltration, low-molecular-weight (MW) hydrolysates had the highest levels of ACE inhibitory (80%), DPPH (84%), RP (0.71 mM trolox equivalents), and FC (81%) activities. Amino acids associated with peptides of high biological activities, such as Val, Ala, Asx, Gly, Lys, Met, Leu, and His, were at higher levels in the low MW fraction compared to any other sample. The biological activities in vitro of all the samples fluctuated under the multiple storage conditions studied, with the highest stability of all the samples appreciated at −20 °C and pH 7. This study shows for the first time the use of giant kelp as a promising source of bioactive peptides and indicates the optimum processing and storing conditions for the use of these compounds as nutraceuticals or functional foods that could help in the prevention of cardiovascular disorders and multiple chronic diseases associated with oxidative damage.

## 1. Introduction

In recent years, brown macroalgae have attracted the attention of researchers as a promising biomass capable of producing a wide variety of chemicals with diverse biological activities and, thus, multiple applications in the food, cosmetic and pharmaceutical industries [1]. Although brown macroalgae are currently commercially exploited mainly as a source of alginate and other hydrocolloids, the biomass also has the potential to be used as a source of proteins, fibres, and lipids, as well as other minor compounds, such as pigments and minerals [2].

Within the group of brown macroalgae, giant kelp (*Macrocystis pyrifera*) is one of the most commercially exploited species due to the fast growth rate of the biomass and, thus, an abundance of raw biomass for the production of high-value compounds. *M. pyrifera* is used as a source of nutrients such as protein, fiber, and minerals, particularly in Asia [3]. Ravanal et al. [4] discovered that the *M. pyrifera* residue following alginate extraction included large amounts of crude protein, which makes this macroalga a good source for protein extraction along with recovering other useful compounds. Moreover, the protein from this macroalga contains all essential amino acids. Li et al. [5] revealed that 35.83% of *M. pyrifera*’s total amino acid contents are made up of essential amino acids.

Despite the possibilities of giant kelp or any other macroalgae to be used as a source of protein, macroalgae currently remain untapped or underutilized biomass as a source of these compounds. Macroalgae have strong cell walls, hindering the extraction of protein and other high-value compounds for their utilization by the food industry [6]. Several studies have previously focused on increasing the extraction of protein from macroalgae by exploring the use of conventional and innovative extraction technologies and/or optimizing extraction conditions to increase the recovery of protein [7,8,9]. Amongst all these innovative technologies, the use of ultrasound-assisted extraction (UAE) gained attention as a more green and efficient method for the recovery of protein and other compounds from macroalgae and a wide variety of other biological matrices [10]. Several studies emphasized that UAE allows the acceleration of the rate of the extraction processes and increases the yields of extraction, resulting in more efficient extraction protocols [11].

Furthermore, once the protein is extracted, the use of several hydrolytic conditions, including the use of solvents and enzymes, have been explored to functionalize proteins into peptides/hydrolysates that can display different biological activities than those described by the original proteins. The use of solvents and strong acids has been linked with serious or uncontrolled damage to proteins [12]. Thus, the use of enzymatic processes with proteases has been gaining momentum to generate controlled hydrolytic conditions that will induce the fast and efficient release of peptides with additional health benefits when used as functional foods [13]. Previous research emphasized the generation of hydrolysates with anti-hypertensive activities in vitro, mainly ACE inhibitory activity from a wide variety of macroalgae [14]. Moreover, protein hydrolysates have excellent potential as antioxidant additives in foods because they can prevent oxidation by different mechanisms [15]. Moreover, purification methods, such as ultrafiltration, are used to separate the compounds of the hydrolysates based on their molecular weight (MW), and these separation techniques also have a strong influence on the anti-hypertensive and antioxidant activities in vitro of various hydrolysates [16,17,18].

Moreover, once these hydrolysates are generated, an in-depth knowledge of the stability of the biological activities of these compounds is also needed, as some of these compounds are meant to be incorporated in several food formulations treated at variable pH and temperature conditions [19,20]. Thus, the processing conditions at which these peptides are biologically active can offer insight into the post-processing techniques needed to preserve these compounds. Thereby, there are some strategies to make the peptides stable in different conditions, such as the encapsulation of the compounds; however, the use of these post-processing techniques increases the cost of manufacturing as well as the final price of the products for consumers [21].

Previous studies on giant kelp focused on the extraction of polysaccharides, such as alginate [22] and fucoidan [23], lipids [24], and in limited instances, protein [25]. However, to our knowledge, no research has yet been conducted examining the impact of ultrasound on protein extraction yield from giant kelp, followed by an analysis of the thermal and ionic stability of different molecular-weight fractions of hydrolysates produced by enzymatic hydrolysis.

This study aims to extract protein by UAE, exploring the main process parameters that will influence protein recovery from giant kelp (*Macrocystis pyrifera*) as well as the hydrolysis and ultrafiltration of the compounds on the basis of the molecular size to explore optimum processing conditions to achieve hydrolysates with high anti-hypertensive (ACE-I inhibitory activity) and antioxidant (DPPH radical scavenging, RP, and FC) activities in vitro. Moreover, the thermal and ionic stability of all the biological properties in vitro of all the hydrolysates were tested by storing these compounds at various temperatures and pHs for 2 weeks, aiming to elucidate the optimum processing conditions of these compounds to maintain their bioactivities intact.

## 2. Materials and Methods

### 2.1. Biological Material and Chemical Reagents

Giant kelp (*M. pyrifera*) powder was purchased from Ecuadorian Rainforest, LLC, Clifton, NJ, USA. Alcalase from *Bacillus licheniformis* (406.80 U/mg) was provided by the National Institute of Genetic Engineering and Biotechnology (Tehran, Iran). All the other reagents used in this study were of analytical grade and purchased from Sigma Aldrich, Inc. (St. Louis, MO, USA).

### 2.2. Proximate Composition Analyses

The dry matter (DM) and ash contents were analyzed using official methods of analysis described by the AOAC.942.05 Horowitz [26]. Protein content was determined using a Kjeldahl apparatus (BUCHI Labortechnik AG, Flawil, Switzerland) with a nitrogen-to-protein conversion factor of 5 [27], and lipid contents were measured using a Soxhlet apparatus as described in Connolly et al. [28]. Briefly, the sample (1 g) was dried and milled using a mortar and pestle. Hexane (100 mL) was used to extract the lipid content over the course of 6 h at 68 °C. A rotary evaporator was used to evaporate the solvent from the extracted lipid. The carbohydrate content was determined according to the method described by Masuko et al. [29] with slight modifications. In summary, 150 µL of concentrated sulfuric acid (98.5%) were added to 30 µL of standards (glucose solutions, 10–50 mg·mL^−1^) or sample. Then, 30 µL of a 5% phenol solution was added to the mixture and shaken for 2 min. The microplate was placed inside a water bath (90 °C, 5 min) and left at room temperature for 10 min. The microplate contents were shaken for 10 min, and the absorbance of the reactions was read at 490 nm using a microplate reader (BioTek, Winooski, VT, USA). The fiber content was determined as described by Lee et al. [30] by adding the sample (1 g) into a capped Duran bottle, followed by the addition of 40 mL of 50 Mm maleate buffer pH 6.0 with 2 mM CaCl_2_ containing porcine pancreatic α-amylase (50 U/mL) and amyloglucosidase (3.4 U/mL). The solutions were incubated at 37 °C for 16 h; the pH was adjusted to pH 8.2, and 0.1 mL of protease derived from *Bacillus licheniformis* (350 U/mL) was added to the mixtures. These mixtures were incubated at 60 °C for 30 min and cooled to room temperature, followed by the addition of 4 volumes of 95% ethanol. The mixtures were stored at room temperature for 1 h and filtered. These filtrates were oven-dried and weighed.

### 2.3. Protein Extraction

The biomass was initially defatted by hexane at a seaweed:solvent ratio of 1:4 (*w*/*v*) and room temperature, as described by Uraipong and Zhao [31]. The defatted seaweed powder was kept at −20 °C until the protein extraction experiments were performed. A conventional protein extraction protocol was applied as described by Fathi et al. [32] with slight modifications. Briefly, defatted seaweed powder was suspended and thoroughly mixed for 4 h in double distilled water (seaweed:solvent ratio of 1:10 *w*/*v*), and the pH of the solutions was adjusted to 10 using 1 M NaOH. Subsequently, the suspensions were centrifuged (5000× *g*, 4 °C, 15 min), and the proteins of the supernatant were further separated by isoelectric precipitation, acidifying the solutions to pH 4.6 using 1 M HCl, followed by centrifugation at the same conditions as described above. The pellets containing proteins were re-suspended in water, neutralized to pH 7, and freeze-dried and preserved at −20 °C for further analyses and processing.

UAE was performed using an ultrasound (US) bath with a frequency of 40 kHz (Beijing Ultrasonic, Beijing, China). UAE experiments were performed using water as a solvent (at a seaweed:solvent ratio of 1:10 *w*/*v*), exploring the effect of this technology using several power levels (50, 100, and 150 W) and extraction times (5, 10, and 15 min). After the sonication process, the mixture samples were treated as mentioned above to extract and separate protein using isoelectric precipitation.

The % of protein recovery of each extraction protocol (conventional/UAE) was estimated using the following equation (Equation (1)).

(1) $$\mathrm{Protein}\;\mathrm{recovery}\;\left(\%\right)=\left[\frac{\mathrm{Protein}\;\mathrm{content}\;\mathrm{of}\;\mathrm{extract}\;\left(g\;\mathrm{protein}\;\mathrm{per}\;g\;\mathrm{extract}\right)\times \;\mathrm{total}\;\mathrm{amount}\;\mathrm{extract}\;\left(g\right)}{\mathrm{Initial}\;\mathrm{protein}\;\mathrm{content}\;\mathrm{of}\;\mathrm{biomass}\;\left(g\;\mathrm{protein}\;\mathrm{per}\;g\;\mathrm{seaweed}\right)}\right]\times 100$$

### 2.4. Enzymatic Hydrolysis and Molecular Weight Cut-Off Filtration

Following the evaluation of the different extraction methods, the most promising extract on the basis of its protein recovery was further hydrolyzed using two proteases (alcalase and proteinase k) following the enzymatic process as described by Mirzapour-Kouhdasht et al. [33]. Briefly, a protein solution with a concentration of 2.5% (*w*/*v*) was prepared in Tris-HCl buffer at pH of 8.5 and 7 for alcalase and proteinase k, respectively. Alcalase (1% of protein weight) was used to initialize the hydrolysis reaction under optimal conditions (80 rpm, 55 °C, 3 h), and proteinase K at the same concentration was then added (80 rpm, 37 °C, 3 h). The enzymatic hydrolysis was stopped by heating the solutions at 100 °C for 20 min. The hydrolysates were fractionated by MW cut-off filtration by using centrifugal ultrafilter units (Millipore, Sigma-Aldrich, St. Louis, MO, USA). The samples were sequentially filtered through units with MW ranging from 1 to 30 kDa, resulting in 5 different fractions: F1 (10 kDa < MW < 30 kDa), F2 (5 kDa < MW < 10 kDa), F3 (3 kDa < MW < 5 kDa), F4 (1 kDa < MW < 3 kDa), and F5 (MW < 1 kDa).

### 2.5. Biological Activities In Vitro

The biological activities in vitro of the full hydrolysate and fractions before and after the application of ionic and thermal stressors was tested in terms of their ACE inhibitory and antioxidant activities (2,2-diphenyl-1-picrylhydrazyl (DPPH) radical scavenging activity, reducing power (RP) activity, and ferrous chelating (FC) activity). All the biological activities in vitro were performed in triplicate.

#### 2.5.1. ACE Inhibitory Activity

The ACE inhibitory activity of the samples was measured following a modified method described by Mirzapour-Kouhdasht et al. [34]. Briefly, 22 µL of ACE (50 mU/mL) was mixed with either 50 µL of samples (1 mg/mL), captopril (positive control, 15 nM), or ACE buffer (negative control). Then, 100 µL of a solution of 0.5 mM furanacryloyl-L-phenylalanylglycylglycine (FAPGG) and 150 µL of ACE buffer (50 mM Tris-HCl, pH 7.5, comprising 0.3 M NaCl and 1 mM ZnCl_2_) were added. The changes in absorbance of the mixtures (Δ) were monitored for 1 h using a microplate reader (BioTek, Winooski, VT, USA) at 340 nm for 1 h. The ACE inhibitory activity of the samples was calculated following the equation as described below (Equation (2)).

ACE inhibitory activity (%) = (1 − ((Δ Absorbance sample)/(Δ Absorbance control))) × 100(2)

#### 2.5.2. DPPH Radical Scavenging Activity

DPPH radical scavenging activities were determined as described by Ambigaipalan and Shahidi [35] with slight modifications. First, 200 μL of samples (1 mg/mL), absolute methanol (negative control) and ascorbic acid (positive control, 100 µg/mL) were added into 800 μL of a 0.1 mM solution of DPPH in 95% methanol. All the mixtures were incubated at room temperature for 30 min, and the absorbance of the reactions was measured using a microplate reader (BioTek Instruments, Winooski, VT, USA) at 517 nm. The DPPH radical scavenging activity of fractions was calculated using the following equation (Equation (3)).

DPPH radical scavenging activity (%) = ((Ac − As)/Ac) × 100(3)

where Ac indicates the absorption of blank samples and As represents the absorption of the samples.

#### 2.5.3. RP Activity

The RP activity of the samples was measured following the method described by Yen and Chen [36]. Samples (1 mg/mL) or standard (trolox, 0–1 mM) were mixed with a 1% potassium ferricyanide solution at a ratio of sample:solution of 1:1 (*v*/*v*), and these mixtures were incubated at 50 °C for 20 min. Following incubation, 2.5 mL of 10% trichloroacetic acid was added, and the mixtures were centrifuged (3000 rpm, 15 min). Following centrifugation, the supernatants were diluted in distilled water (1:1, *v*/*v*). Then, 0.5 mL of a ferric chloride solution (1%, *w*/*v*) was added to each mixture, and the solutions were thoroughly mixed and incubated at 25 °C for 10 min. The absorbance of the mixtures was read at 700 nm in a spectrophotometer (Perkin-Elmer UV–VIS-NIR, Waltham, MA, USA). The RP of the samples was expressed as mM trolox equivalents.

#### 2.5.4. FC Activity

The FC activity of the samples was determined according to Ambigaipalan et al. [37], with slight modifications. First, 200 µL of samples (1 mg/mL) or distilled water (blank) were diluted into 1.74 mL of distilled water and mixed with 40 µL of 5 mM ferrozine and 20 µL of 2 mM FeCl_2_. The mixtures were thoroughly mixed and incubated at 25 °C (10 min), and the absorbance of the reactions was measured using a Perkin-Elmer UV–VIS-NIR spectrophotometer at 562 nm. FC was calculated following the equation as described below (Equation (4)).

FC (%) = [(1 − As)/Ac)] × 100(4)

As and Ac relates to the absorbance of the samples and control, respectively.

### 2.6. Amino Acid Composition

The amino acid composition of the full hydrolysate and fractions was determined as described in Mirzapour-Kouhdasht, Moosavi-Nasab, Krishnaswamy and Khalesi [16]. In short, 50 mg were hydrolyzed using 6 M HCl with 0.1% phenol at 110 °C for 24 h. Norleucine (Sigma Aldrich, Inc., St. Louis, MO, USA) was used as an internal standard for the calibration. The amino acid content of the solutions was measured by an amino acid analyzer (HITACHI 8900 Amino Acid Analyzer, Tokyo, Japan). All the samples were analyzed in triplicate.

Several calculations were made to elucidate: (1) essential amino acid values by adding the concentrations of His, Ile, Leu, Lys, Met, Phe, Thr, and Val; (2) conditionally essential amino acid concentrations by adding the values of Arg, Gly, Pro and Tyr; (3) non-essential amino acids values that correspond to Ala, Asx, Glx, Cys, and Ser; (4) and total amino acids that are calculated by adding all the previous values.

### 2.7. Thermal and Ionic Stability of Hydrolysates

The thermal and ionic stability of full hydrolysate and fractions was determined as described by Krungkri and Areekul [38]. Briefly, the thermal stability of the samples was tested by storing the freeze-dried samples at various temperatures (−20, 4, 37 and 60 °C) at controlled humidity for 2 weeks. The ionic stability of the compounds was tested by mixing lyophilized samples at a ratio of 1:1 (*w*/*v*) with solutions sterilized by autoclave (121 °C, 20 min) of various pHs (2, 5, 7, 9 and 11). All the samples were kept in sterile tubes to prevent any microbial intervention in the experiment. 

### 2.8. Statistical Analyses

All statistical analyses were performed using SPSS version 23 (IBM, North Castle, NY, USA). Differences were analyzed using multivariate general linear models, and the differences were analyzed further by Tukey’s Honest Significant Difference (HSD) post hoc tests. In all cases, the criterion for statistical significance was *p* < 0.05.

## 3. Results and Discussion

### 3.1. Proximate Composition

The proximate composition of the giant kelp used in all the extraction experiments is summarized in Table 1.

Overall, the proximate composition of the macroalgal biomass used in this study agrees with previous reports in the literature. Similar to the results of this study, Cuesta-Gomez and Sánchez-Saavedra [39] reported protein contents of 10.50% in *M. pyrifera*. The harvesting season and region of the collection can also play a role in the variable proximate composition data currently available for this macroalga [27,40]. Thereby, the carbohydrate contents of the giant kelp powder in this study were lower than the 40% previously described by Navarrete et al. [41]; however, Mansilla and Ávila [40] reported variable carbohydrate contents of giant kelp ranging between 3.27 and 8.46%, similar to those described in the current study. Similarly, these authors also described levels in the range of 0.4–0.84%, 29.88–37.18%, and 14.58–20.43% for lipid, ash, and fiber content in giant kelp depending on the harvesting season, which relates well to the proximate composition of the current study [40].

### 3.2. Protein Extraction

**Figure 1:**
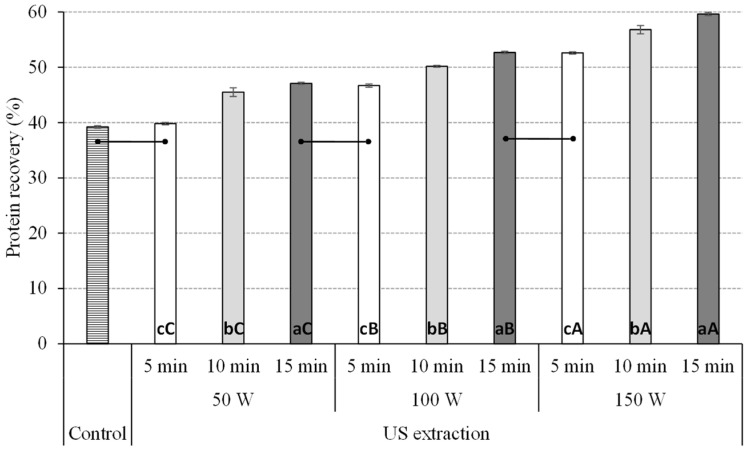
Effect of extraction conditions time (5, 10 and 15 min) and ultrasonic (US) power (50, 10 and 150 W) on the protein recovery from giant kelp. Data are presented as average ± standard deviation (*n* = 3). Bars inside the figure indicate extraction conditions showing no statistical differences in terms of protein recovery for all the extraction conditions tested. Different lowercase letters represent statistical differences (*p* < 0.05) in protein recovery at different times of extraction using the same ultrasound (US) power, while different uppercase letters represent statistical differences (*p* < 0.05) in protein recovery at different US power using the same extraction time.

The protein recovery (%) achieved by several combinations of UAE using variable extraction times (5, 10 and 15 min) and ultrasonic (US) power (50, 100 and 150 W) compared to control experiments (conventional extraction, 4 h) are shown in Figure 1.

Overall, the application of UAE had a favorable effect in increasing the protein recovery yields with respect to control conditions. This confirms previous reports on the benefits of the use of UAE to improve the recovery of protein from alternative protein sources, as well as other improvements in extraction processes as reviewed by Das, Tiwari, Chemat, and Garcia-Vaquero [10]. The conventional method used as control achieved protein recoveries of 39.22%, similar to those achieved when using UAE at 50 W for 5 min (39.82%); however, the application of any other UAE condition achieved higher protein recovery yields, reaching the highest recovery yields (approximately 60%) when using UAE at 150 W for 15 min. There was a statistically significant influence of the extraction time, ultrasonic power and the interaction of these parameters (*p* < 0.001) on the protein recovery from giant kelp. Overall, as seen in Figure 1, an increased extraction time and ultrasonic power had a positive effect when increasing the protein recovery from giant kelp. To our knowledge, no research has previously reported the recovery of protein from giant kelp using UAE. However, our results agree with previous research using UAE to recover protein from another algal biomass. Thereby, Hildebrand et al. [42] explored UAE combined with an alkaline solvent to recover protein from *Chlorella vulgaris*. The authors reported that the UAE method resulted in a protein recovery 1.32-fold higher than the conventional method without the use of ultrasounds. Kadam, Álvarez, Tiwari and O’Donnell [8] reported similar results to those of this current study when examining the effect of US on the extraction of protein from *Ascophyllum nodosum*. The authors illustrated that a single step of 0.1 M alkali extraction assisted with US (68.4 μm amplitude) increased the recovery of protein extraction, reaching levels of 57%, significantly higher than those achieved when using US at 22.8 μm with an amplitude of approximately 26%. Moreover, the influence of time when using UAE was also previously reported in previous studies. I.e. Mittal et al. [43] indicated that increasing the time of US extraction from 2 to 10 min while keeping using a fixed US amplitude of 120 µm resulted in increases of protein extraction from *Gelidium pusillum* from approximately 0.04 mg protein/g dry matter to reaching levels of 0.16 mg protein/g dry matter.

### 3.3. Amino Acid Composition of Protein Hydrolysates

Protein extraction conditions achieving the highest protein recovery from giant kelp (UAE, 150 W, 15 min) were selected as the optimum conditions for further processing. The protein generated using these conditions was hydrolyzed, and the full hydrolysates were fractionated by ultrafiltration to generate several fractions of variable MW. The fractions generated were F1 (30 kDa > MW > 10 kDa), F2 (10 kDa > MW > 5 kDa), F3 (5 kDa > MW > 3 kDa), F4 (3 kDa > MW > 1 kDa), and F5 (MW < 1 kDa). The amino acid contents of the full hydrolysate and the ultrafiltered fractions are summarized in Table 2.

The results of the amino acid analysis indicate that the critical amino acids in all fractions were Asx, Glx, Val and Met. Machado et al. [44] also reported that the amino acid composition of a red macroalga (*Porphyra* sp.) contained high levels of free Ala, Glu, and Asp, while Met and Trp were the main limiting amino acids of this macroalga. When comparing the amino acid profile of all the samples, F5 (MW < 1 kDa) had a higher content of each of the analyzed amino acids when compared to other fractions and even the full hydrolysate, except in the case of Gly and Cys, which were at its highest in F3 and F4, and Tyr and Phe, which were at their maximum in F3. Similarly, Mirzapour-Kouhdasht, Moosavi-Nasab, Krishnaswamy, and Khalesi [16] also reported higher contents of Gly, Pro and Ala in low-MW fractions generated from barred mackerel gelatin hydrolysates.

When analyzing the nutritional value of these hydrolysates, the total essential amino acids (His, Ile, Leu, Lys, Met, Phe, Thr, Val), conditionally essential amino acids (Arg, Gly, Pro, Tyr) and non-essential amino acids (Ala, Asx, Glx, Ser) were calculated as seen in Table 2. F5 was also the fraction containing the highest content of essential and non-essential amino acids in comparison to other fractions and full hydrolysate. Thus, this fraction will be nutritionally relevant when using these compounds as new ingredients in food formulations. Pimentel et al. [45] also reported high levels of protein, up to 47% dry weight, with high levels of all essential amino acids in several macroalgal species. There are few reports on the amino acid composition of macroalgal protein hydrolysates. Paiva et al. [46] reported the amino acid composition of different MW fractions of protein hydrolysates derived from *Fucus spiralis*. The authors also reported that the low MW fraction (<1 kDa) had the highest content of essential amino acids of 1.8 g/100 g DW macroalgae. As for the conditionally essential amino acids, there was no substantial difference in their content between F5, F4, F3, and F2, but all these fractions had higher levels of these amino acids compared to F1 and the full hydrolysate. In a study conducted by Purcell et al. [47], it was reported that the sum of conditionally essential amino acids obtained from *Laminaria digitata* decreased from 2.7 g/100 g DW to 1.5 g/100 g DW after passing through an ultrafiltration unit with the molecular weight cut off 3 kDa. This depletion of some amino acids could be due to the inability of some peptides containing them to have a molecular weight higher than the filter cut-off [44].

### 3.4. Biological Activities In Vitro

Enzymatic hydrolysis and ultrafiltration generate differences in the amino acid profiles of the different fractions that may affect the biological activities in vitro of the hydrolysates. These activities are summarized in Table 3.

Overall, the fractions with low MW had increased levels of ACE inhibitory activity, DPPH radical scavenging activity, RP activity, and FC activity.

Blood pressure is regulated by the renin-angiotensin system, in which ACE converts the inactive angiotensin I into angiotensin II with a potent vasoconstriction effect. Currently, there are synthetic drugs used as ACE inhibitors; however, their use has been linked to undesirable side effects. Thus, research on natural sources of ACE inhibitors, such as the macroalgal hydrolysates described in the current study, can be a promising alternative to these drugs to regulate blood pressure. In this study, the fractionation of the full macroalgal hydrolysate into multiple fractions of different MW had a significant influence on the ACE inhibitory activities of the hydrolysate that reached maximum levels in the fraction of the lowest MW F5 with ACE inhibitory activity of approximately 3-fold higher than those described in the full hydrolysate. These results are in agreement with previous studies reporting a higher ACE inhibitory activity in low MW peptides [48]. Liu, Zhang, Miyakawa, Li, Gu, and Tanokura [48] also reported that when generating peptides from food proteins, higher ACE inhibitory activities are expected in short peptides with MW of less than 1000 kDa. Moreover, Moayedi, et al. [49] also demonstrated that the presence of peptide fragments in low-MW fractions rich in certain amino acid residues, such as Val, Ala and Tyr, similar to the ones of the current study, was associated with high ACE inhibitory activities.

Similarly to the case of ACE inhibitory activities, the antioxidant activities in vitro of the hydrolysates increased in fractions containing low-MW compounds, with the fraction F5 having levels approximately 4, 7, and 6-fold higher compared to those of the full hydrolysate’s DPPH radical scavenging, RP and FC activities, respectively. Previous studies also found that the antioxidant activities evaluated by various antioxidant in vitro assays of peptides from hydrolysates depend on the MW of the compounds [50,51,52]. Researchers hypothesized that low-MW protein hydrolysates and peptides could serve better as electron donors and react with free radicals to transform them into stable substances compared to high-MW peptides [53]. Tkaczewska, Borawska-Dziadkiewicz, Kulawik, Duda, Morawska, and Mickowska [53] also reported that compounds with high RP have a high ability to donate electrons or hydrogen and serve as a significant indicator of use as an antioxidant. The amino acid composition of the fractions of low MW of this study, high in hydrophobic amino acids, especially the higher contents of Lys, Met, Leu, Tyr, His, Trp and Ile, can be related to the high RP of the hydrolysates as previously described by Qian et al. [54].

### 3.5. Thermal and Ionic Stability of Hydrolysates

The stability of bioactive peptides and their biological activities, particularly to multiple heat and pH treatments used in food formulations, is relevant for this study as thermal and ionic conditions may have a huge effect and lead to the loss of biological activities of the compounds [38]. Consequently, the bioactivity of these compounds should be maintained during the shelf-life and storage of the food products to which they are added to.

**Figure 2:**
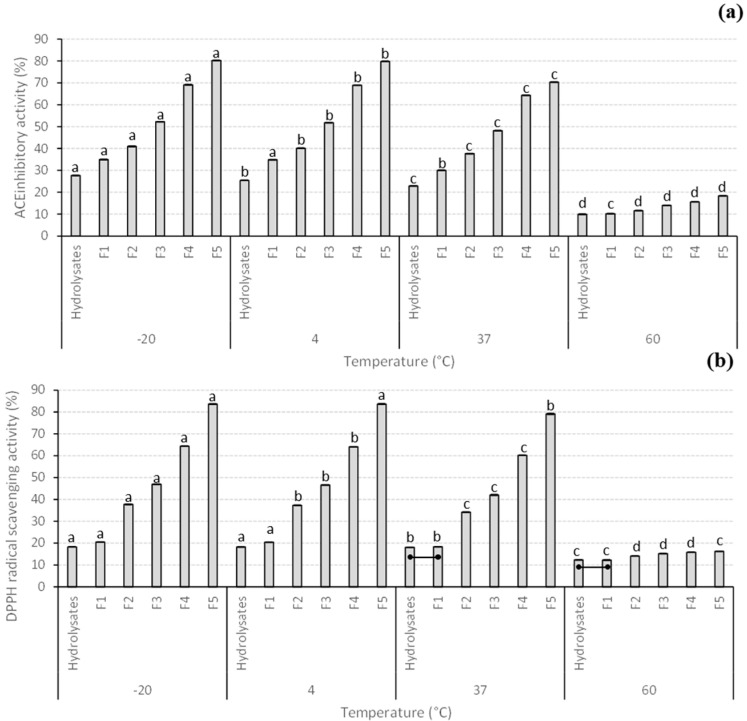

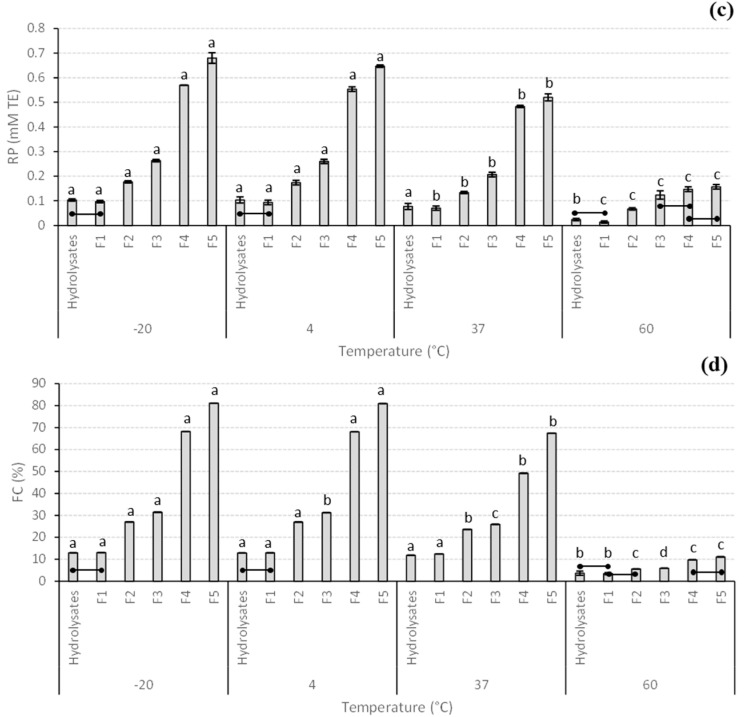
Stability of the biological activities in vitro (**a**) ACE inhibitory activity (%), (**b**) DPPH radical scavenging activity (%), (**c**) RP activity (mM TE) and (**d**) FC activity (%) of giant kelp protein hydrolysates and different MW fractions at various temperatures (−20, 4, 37 and 60 °C). Data are presented as the average ± standard deviation (*n* = 3). Different lowercase letters indicate statistical differences (*p* < 0.05) in the biological activities in vitro of the same sample type at different temperatures. Lines inside the figure join statistically similar (*p* > 0.05) biological activities in vitro between different samples tested at the same temperature. Abbreviations in the figure are as follows: F1 (30 kDa > MW > 10 kDa), F2 (10 kDa > MW > 5 kDa), F3 (5 kDa > MW > 3 kDa), F4 (3 kDa > MW > 1 kDa), and F5 (MW < 1 kDa).

The effects of different temperatures (−20, 4, 37, and 60 °C) for 2 weeks on the biological activities in vitro of the full hydrolysate and ultrafiltered fractions generated from giant kelp are summarized in Figure 2. There were statistical differences in the stability of all biological activities tested depending on the type of sample, temperature, and the interaction between both factors. Overall, as seen in Figure 2, the highest levels of all biological activities in vitro and all the fractions tested were at −20 °C/4 °C, followed by 37 °C, and reaching a minimum at 60 °C. Moreover, F5 had the highest levels of all the biological activities tested in vitro at all the temperatures considered in this study. F5 had the highest levels of ACE inhibitory activity at −20 °C with a slight but statistically significant decrease in ACE inhibitory activity at 4 and 37 °C. The ACE inhibitory activity reached a minimum, with levels < 20% ACE inhibition, when preserved at 60 °C. In the case of DPPH, RP, and FC activities, F5 was highly stable and with no statistical differences when tested at −20 and 4 °C, followed by a significant decrease at 37 °C that reached minimum levels at 60 °C. These results were expected as heat causes protein denaturation and aggregation over time, which may cause the high-molecular-mass peptides to form clusters, impeding their binding ability to enzymes, such as ACE, explaining this loss in ACE inhibitory activity and other bioactivities [55]. Moreover, previous studies also reported that heat treatments can damage specific amino acids related to the ACE inhibitory activity of peptides, also disrupting the structure of some peptides that can also affect their ACE inhibitory activity [56]. Geng et al. [57] evaluated the ACE inhibitory activity of *Tricholoma matsutake* peptide (WALKGYK) at temperatures between 40 and 90 °C and reported that the lowest temperature tested (40 °C) was the best one to preserve the biological activity of these fractions. On the other hand, the hydrolysis of peptide bonds as a result of very high or low pH values, especially at the temperature of 60 °C, could also be the reason for the reduction in the biological activity of samples during the stability test [55]. To our knowledge, there are limited reports available on the influence of temperature on the preservation of the antioxidant activities in vitro of protein hydrolysates from different sources.

**Figure 3:**
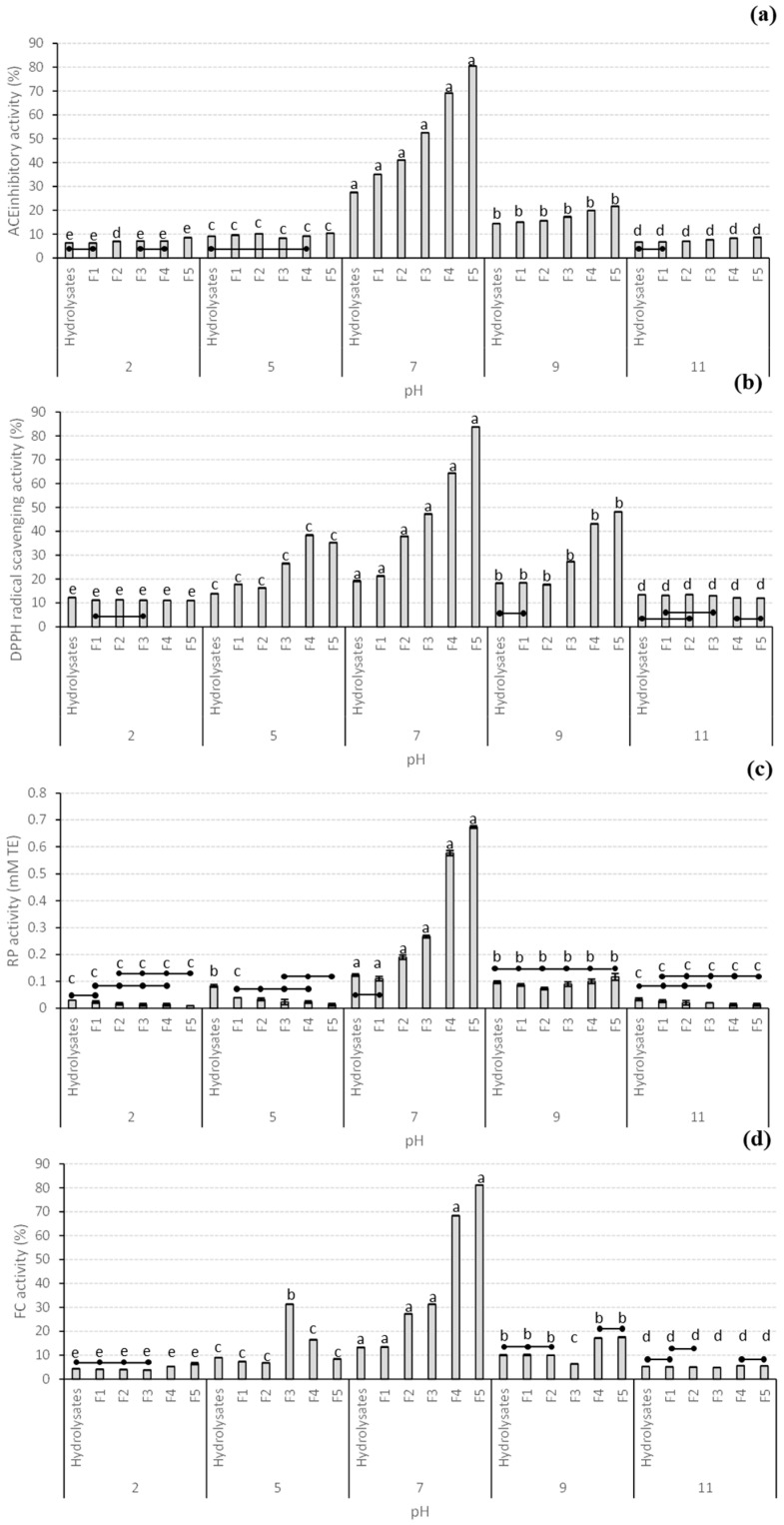
Stability of the biological activities in vitro (**a**) ACE inhibitory activity (%), (**b**) DPPH radical scavenging activity (%), (**c**) RP activity (mM TE) and (**d**) FC activity (%) of giant kelp protein hydrolysates and different MW fractions at various pH (2–11). Data are presented as the average ± standard deviation (*n* = 3). Different lowercase letters indicate statistical differences (*p* < 0.05) in the biological activities in vitro of the same sample type at different pHs. Lines inside the figure join statistically similar (*p* > 0.05) biological activities in vitro between different samples tested at the same pH. Abbreviations in the figure are as follows: F1 (30 kDa > MW > 10 kDa), F2 (10 kDa > MW > 5 kDa), F3 (5 kDa > MW > 3 kDa), F4 (3 kDa > MW > 1 kDa), and F5 (MW < 1 kDa).

The ionic stability of the hydrolysate and all different MW fractions and their ability to retain their biological activities in vitro were tested at various pHs (2, 5, 7, 9, and 11), as seen in Figure 3. Similar to the case of thermal stability, all the biological activities in vitro varied depending on the type of sample, the pH level, and interactions between these 2 factors. Contrary to the case of thermal stability, the biological activities in vitro of the hydrolysates were extremely sensitive to changes in pH, with all the biological activities in vitro being at their highest levels at pH 7 for all the fractions, with the fraction F5 also displaying the maximum values for all these bioactivities. Increases and decreases in pH beyond 7 resulted in dramatic reductions in ACE and RP activities, which were always beyond 25% and 0.15 mM TE for ACE and RP, respectively. In the case of DPPH and FC activities, pHs 5 and 9 also resulted in a decreased level of both activities in all the fractions; however, some fractions still displayed significantly higher antioxidant power compared to when tested at pHs 2 and 11. Similarly to the results of this study, Geng, Tian, Zhang, Zhao, Zhao, Wang, and Ng [57] also reported that the ACE inhibitory activity of *Tricholoma matsutake* peptide at pH 6 was higher compared to other pH levels tested that ranged from 2 to 11. Several reports mentioned that at neutral pH, the antioxidant activity of peptides is high, decreasing significantly under per-acidic or alkaline conditions [58,59]. This fact could be attributed to the interaction between hydrogen and hydroxyl groups of the charged regions of peptides, leading to the breakage of the hydrogen bonds between amino acids that will ultimately generate peptide denaturation [60]. Moreover, ionic changes have also been reported as displaying direct damage to several amino acid residues responsible for some of the biological activities of peptides, with acidic treatments inducing damage to Glu and Asn and alkaline treatments responsible for the alteration of Cys, Ser, and Thr amino acid residues [55].

## 4. Conclusions

Overall, US extraction (150 W, 15 min) was the most effective method for the generation of protein isolates from giant kelp for further processing for the generation of hydrolysates. When generating hydrolysates, ultrafiltration resulted in a significant change in the amino acid content of the fractions that were also reflected in higher anti-hypertensive and antioxidant activities in vitro in all the fractions generated, particularly the low-MW fraction, compared to the original hydrolysate. In terms of the stability of the compounds generated in this study, the hydrolysate and all the generated fractions had moderate thermal stability, with antioxidant activities of all the fractions still active at temperatures ranging from −20 to 37 °C, while the anti-hypertensive activities of the compounds required their preservation at −20 °C. All the compounds had low ionic stability, with all their biological activities preserved at pH 7 and then decreasing at any other tested pH (2, 5, 9 and 11), although some of the antioxidant activities (DPPH and FC) were still retained by the hydrolysates at pHs 5 and 9. To our knowledge, this is the first study exploring the generation of protein hydrolysates with biological activities from giant kelp. The results of this study indicate that low-MW hydrolysates generated from giant kelp can be used as promising food ingredients with anti-hypertensive and antioxidant activities in vitro that could be used directly in multiple food formulations. Future research is needed in these fractions in order to fully elucidate the MW of the most biologically active peptides by size-exclusion chromatographic methods, as well as the elucidation of the sequence of these peptides by mass spectrophotometry. Moreover, further studies are required in order to induce further changes in these compounds to increase their thermal and ionic stability to allow their widespread utilization in the food industry as well as future in vivo and/or ex vivo studies to confirm the biological activities in vitro described in the current study.

## Figures and Tables

**Table 1:** Proximate composition of dried giant kelp (*M. pyrifera*). Units in the table are in g compounds per 100 g dry weight (DW) macroalgal powder. Results are expressed as average ± standard deviation (*n* = 3).

Dry Matter (g/100 g)	Protein Content (g/100 g)	Carbohydrate Content (g/100 g)	Crude Fat (g/100 g)	Ash Content (g/100 g)	Fiber Content (g/100 g)
91.02 ± 0.13	11.42 ± 0.44	17.14 ± 0.15	1.57 ± 0.03	38.05 ± 0.03	22.82 ± 0.19

**Table 2:** Amino acid composition of full hydrolysate of giant kelp and its ultrafiltered fractions of different MW.

Amino Acid Residues *	Hydrolysate	F1	F2	F3	F4	F5
**Essential amino acids**						
His	161.80 ± 0.25 ^e^	162.01 ± 0.02 ^e^	175.16 ± 0.03 ^d^	179.28 ± 0.01 ^c^	182.01 ± 0.04 ^b^	197.43 ± 0.01 ^a^
Ile	511.22 ± 2.51 ^f^	514.59 ± 0.01 ^e^	523.19 ± 0.01 ^d^	530.41 ± 0.03 ^c^	539.13 ± 0.05 ^b^	548.05 ± 0.06 ^a^
Leu	370.88 ± 1.34 ^f^	376.18 ± 0.02 ^e^	382.22 ± 0.03 ^d^	391.03 ± 0.03 ^c^	399.41 ± 0.04 ^b^	408.27 ± 0.02 ^a^
Lys	309.88 ± 1.6 ^f^	318.06 ± 0.08 ^e^	325.69 ± 0.01 ^d^	331.69 ± 0.03 ^c^	342.13 ± 0.04 ^b^	353.17 ± 0.01 ^a^
Val	1150.33 ± 3.40 ^e^	1158.79 ± 0.02 ^d^	1165.20 ± 0.04 ^c^	1168.35 ± 0.04 ^c^	1174.68 ± 0.37 ^b^	1182.25 ± 0.01 ^a^
Met	1121.37 ± 2.37 ^f^	1130.47 ± 0.02 ^e^	1134.82 ± 0.02 ^d^	1141.55 ± 0.01 ^e^	1148.16 ± 0.005 ^b^	1153.10 ± 1.76 ^a^
Thr	733.07 ± 1.80 ^e^	739.41 ± 0.10 ^d^	743.55 ± 1.70 ^c^	749.76 ± 0.11 ^b^	756.12 ± 0.02 ^a^	757.07 ± 0.12 ^a^
Phe	593.95 ± 3.51 ^d^	599.16 ± 0.01 ^c^	608.16 ± 0.05 ^b^	617.37 ± 0.02 ^a^	598.15 ± 0.04 ^c^	597.68 ± 0.24 ^cd^
Total essential amino acids	4952.53 ± 5.7 ^f^	4998.70 ± 0.16 ^e^	5058.02 ± 1.71 ^d^	5109.48 ± 0.15 ^c^	5139.82 ± 0.18 ^b^	5197.04 ± 1.49 ^a^
**Conditionally essential amino acids**						
Arg	950.55 ± 0.18 ^f^	967.20 ± 0.12 ^e^	971.27 ± 0.03 ^d^	978.57 ± 0.07 ^c^	986.63 ± 0.34 ^b^	997.32 ± 0.28 ^a^
Pro	0.95 ± 0.01 ^f^	1.17 ± 0.005 ^e^	1.25 ± 0.01 ^d^	1.38 ± 0.01 ^c^	1.49 ± 0.01 ^b^	1.69 ± 0.01 ^a^
Gly	669.90 ± 1.99 ^b^	675.06 ± 0.08 ^ab^	683.83 ± 0.08 ^ab^	690.20 ± 0.11 ^a^	691.43 ± 0.29 ^a^	679.80 ± 16.78 ^ab^
Tyr	429.29 ± 2.32 ^d^	438.15 ± 0.03 ^c^	446.52 ± 0.02 ^b^	451.04 ± 0.02 ^a^	431.80 ± 0.02 ^d^	431.73 ± 0.03 ^d^
Total conditionally essential amino acids	2050.71 ± 6.08 ^c^	2081.59 ± 0.03 ^b^	2102.88 ± 0.09 ^a^	2121.19 ± 0.18 ^a^	2111.36 ± 0.49 ^a^	2110.55 ± 0.70 ^a^
**Non-essential amino acids**						
Asx	1336.32 ± 1.05 ^e^	1337.29 ± 0.01 ^e^	1345.28 ± 0.10 ^d^	1353.21 ± 0.03 ^c^	1361.09 ± 0.07 ^b^	1380.44 ± 0.005 ^a^
Glx	1822.74 ± 2.16 ^d^	1831.64 ± 0.34 ^c^	1833.54 ± 0.36 ^bc^	1834.36 ± 0.16 ^b^	1837.42 ± 0.51 ^a^	1837.66 ± 0.14 ^a^
Ser	830.49 ± 3.37 ^e^	834.44 ± 0.15 ^d^	839.22 ± 0.19 ^c^	848.01 ± 0.05 ^b^	851.15 ± 0.12 ^b^	855.60 ± 0.03 ^a^
Ala	642.99 ± 1.50 ^f^	650.31 ± 0.08 ^e^	657.37 ± 0.01 ^d^	661.84 ± 0.01 ^c^	676.35 ± 0.14 ^b^	690.15 ± 0.01 ^a^
Cys	207.46 ± 0.89 ^d^	208.17 ± 0.02 ^d^	219.16 ± 0.16 ^b^	229.23 ± 0.09 ^a^	230.16 ± 0.02 ^a^	209.54 ± 0.18 ^c^
Total non-essential amino acids	4840.00 ± 2.57 ^f^	4861.82 ± 0.81^e^	4894.57 ± 0.97 ^d^	4926.65 ± 0.68 ^c^	4956.19 ± 0.72 ^b^	4973.40 ± 0.42 ^a^
**Total amino acid residues**	**11843.26 ± 10.18 ^f^**	**11942.12 ± 0.45^e^**	**12055.48 ± 1.36 ^d^**	**12157.34 ± 0.26 ^c^**	**12207.38 ± 0.99 ^b^**	**12281.00 ± 17.64 ^a^**

* Amino acid residues are reported as mg/100 g DW hydrolysate. Data in the table are presented as average ± standard deviation (*n* = 3). Different letters within each row represent statistical differences between different fractions in the content of each individual amino acid (*p* < 0.05). Abbreviations in the table are as follows: F1 (30 kDa > MW > 10 kDa), F2 (10 kDa > MW > 5 kDa), F3 (5 kDa > MW > 3 kDa), F4 (3 kDa > MW > 1 kDa), and F5 (MW < 1 kDa).

**Table 3:** Biological activities in vitro of full hydrolysate of giant kelp and its ultrafiltered fractions of different MWs.

Biological Activities In Vitro	Hydrolysate	F1	F2	F3	F4	F5
ACE inhibitory activity (%)	27.60 ± 0.005 ^f^	35.37 ± 0.17 ^e^	41.08 ± 0.05 ^d^	52.68 ± 0.04 ^c^	70.21 ± 0.98 ^b^	80.46 ± 0.02 ^a^
DPPH radical scavenging activity (%)	19.36 ± 0.09 ^f^	21.36 ± 0.01 ^e^	38.03 ± 0.03 ^d^	47.29 ± 0.01 ^c^	64.77 ± 0.02 ^b^	83.93 ± 0.02 ^a^
RP activity (mM TE)	0.10 ± 0.01 ^e^	0.11 ± 0.01 ^e^	0.18 ± 0.005 ^d^	0.28 ± 0.00 ^c^	0.59 ± 0.01 ^b^	0.71 ± 0.01 ^a^
FC activity (%)	13.57 ± 0.3 ^e^	13.63 ± 0.04 ^e^	27.30 ± 0.01 ^d^	31.63 ± 0.03 ^c^	68.48 ± 0.03 ^b^	81.28 ± 0.1 ^a^

All samples were tested at 1 mg·mL^−1^. Data are presented as average ± standard deviation (*n* = 3). Different letters within each row represent statistical differences between different fractions (*p* < 0.05). Abbreviations in the table are as follows: F1 (30 kDa > MW > 10 kDa), F2 (10 kDa > MW > 5 kDa), F3 (5 kDa > MW > 3 kDa), F4 (3 kDa > MW > 1 kDa), and F5 (MW < 1 kDa).

## Data Availability

All data generated or analyzed during this study are included in this published article.
